# Explainable machine learning approach for cancer prediction through binarilization of RNA sequencing data

**DOI:** 10.1371/journal.pone.0302947

**Published:** 2024-05-10

**Authors:** Tianjie Chen, Md Faisal Kabir

**Affiliations:** Department of Computer Science, Pennsylvania State University Harrisburg, Middletown, Pennsylvania, United States of America; Abdul Wali Khan University Mardan, PAKISTAN

## Abstract

In recent years, researchers have proven the effectiveness and speediness of machine learning-based cancer diagnosis models. However, it is difficult to explain the results generated by machine learning models, especially ones that utilized complex high-dimensional data like RNA sequencing data. In this study, we propose the binarilization technique as a novel way to treat RNA sequencing data and used it to construct explainable cancer prediction models. We tested our proposed data processing technique on five different models, namely neural network, random forest, xgboost, support vector machine, and decision tree, using four cancer datasets collected from the National Cancer Institute Genomic Data Commons. Since our datasets are imbalanced, we evaluated the performance of all models using metrics designed for imbalance performance like geometric mean, Matthews correlation coefficient, F-Measure, and area under the receiver operating characteristic curve. Our approach showed comparative performance while relying on less features. Additionally, we demonstrated that data binarilization offers higher explainability by revealing how each feature affects the prediction. These results demonstrate the potential of data binarilization technique in improving the performance and explainability of RNA sequencing based cancer prediction models.

## Introduction

Machine learning (ML) models have been used for cancer research for almost 40 years. In the past, researchers primarily focused on using clinical and demographic data to individual’s risk of developing cancer [1]. Recent advancements in genomic and computational technology has enabled researchers to study cancer more thoroughly and develop new models for cancer prediction and survival analysis [2–5].

One proven way to study cancer using computational and ML-based methods is by analyzing peptides, specifically anti-cancer peptides (ACPs) data. Because of their low toxicity and greater efficacy, ACPs have recently attracted researchers’ interests as a promising therapeutic agent for cancer treatment. However, efficient identification of ACPs is still a challenge. To address this issue, researchers have proposed multiple ML-assisted tools for prediction of ACPs. Some early examples of utilizing ML models such as support vector machine (SVM) to identify ACPs are Chou’s pseudo-amino acid composition (PseAAC) and sequence-based identification of anticancer peptides (iACP) [6, 7]. These methods paved the foundation for approaches that incorporated more advanced ML techniques like feature selection, dimensionality reduction, ensemble learning, and genetic algorithm. To further improve the performance of iACP method, a novel model called iACP-GAEnscC was developed based on ensemble learning and evolutionary genetic algorithm [8]. Dimensionality reduction techniques like principal component analysis (PCA) was also used to develop effective models like cACP for prediction of ACPs [9]. In particular, the cACP model was further developed into cACP-2LFS model, which utilized a two-level feature selection method to improve performance on existing models, and cACP-DeepGram model, which achieved better performance by incorporating a FastText-based word embedding strategy to represent each peptide sample [10, 11].

Another proven way is to utilize patients’ RNA sequencing (RNA-seq) data. However, using RNA-seq data for cancer prediction poses a challenge to researchers because of their high dimensionality, complexity, and redundancy, which could lead to decrease in accuracy and efficency [12]. To combat this issue, researchers utilized dimensionality reduction and feature selection methods, such as univariate feature selection [13, 14], stepwise feature selection [15], PCA [16], autoencoder [17–19], and hybird approaches [20]. However, both feature selection and dimensionality reduction have some hard-to-fix drawbacks. Because feature selection functions by extracting a subset of features that is more related to the label, it ignores the inter-relationships between features [21]. On the other hand, interpreting new data of a lower dimension generated by dimensionality reduction techniques like PCA and autoencoder is difficult because one new feature could correlate to multiple original features [22].

Interpreting a ML model has always been a difficult task. Fundamentally, ML models can be divided into two groups: white box models and black box models. White box models like decision tree and logistic regression are easiler to interpret because they have built-in feature importance that explains how each feature contributes to predictions [23]. Black box models, on the other hand, must rely on post-hoc explanation approaches. A few popular post-hoc explanation techniques are Local interpretable model-agnostic explanations (LIME) [24], SHapley Additive exPlanations (SHAP) [25], saliency map, and counterfactual explanations. Although existing peptides and RNA-based models have shown encourging performances in producing accurate predictions, they are limited in terms of interpretability. Therefore, researchers have developed several models to address this issue. For peptides-based models, both white box and post-hoc explanation techniques were used. White box models like ACPred used rule extraction on random forest models to extract decision rules [26]. On the other hand, post-hoc explanation like LIME and SHAP are the preferred choice for explaining more complexed models that are based on neural networks or ensemble learning like iAFPs-EnC-GA, AIPs-SnTCN, and ACPred-BMF [27–29]. For RNA-based models, most studies chose post-hoc explanations like SHAP because of the complexity of the algorithms used [30, 31]. Despite having many choices, interpreting models that use continuous features is still difficult. For continuous features, most explanation techniques that generate feature importance scores only shows the names of importance features and how each of them contributes to predictions in terms of importance-like scores. This kind of interpretation is not meaningful as users would not understand how each feature, in terms of its original value, contributes to predictions. To combat this issue, researchers proved that, by binarilizing continuous features, interpreting ML models become much easier as each feature is meaningful [32].

Data binarilization has already been proven to be able to increase the interpretability of ML models while maintaining predictive accuracy. Similar approach has been used to identify ACPs and demonstrated its effectiveness [33]. However, this technique has never been applied to RNA-seq data before. Therefore, we propose a data binarilization-based approach to increase the performance and interpretability of ML models used for cancer prediction.

The contributions of this study are:

- Data binarilization technique is proposed for processing RNA-seq data.
- Multiple models were constructed and tested to examine the effectiveness of the proposed technique.
- Models using proposed technique were compared with models using other state-of-the-art techniques.
- Models using proposed technique showed promising results.
- Proposed technique provides easier-to-understand explanations.

This paper is divided into five sections. Section one consists of reviews on the use of ML models in cancer prediction and ML techniques on interpretability. Section two delineates the methodology of the study, including data collection, data binarilization, feature selection, classification model, hyperparameter search, performance metrics, and model explanaation. Re- sults and analyses are located in section three. Section four discusses the advantages of our proposed technique, contributions and limitations of this study, as well as future research plans. Finally, sections five concludes this paper by providing an overview of this study and future research directions.

## Materials and methods

**Fig 1:**
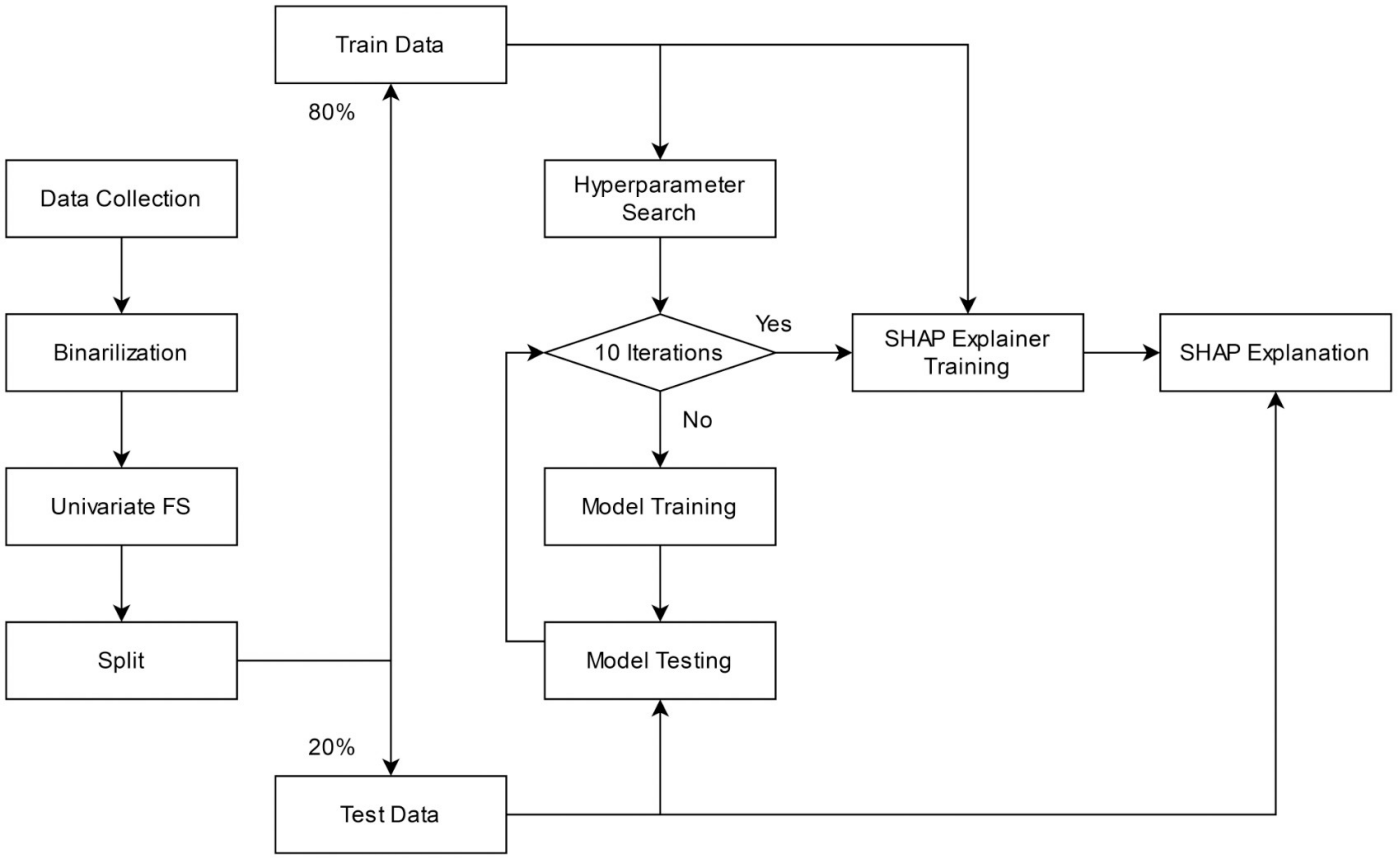
Flowchart of the proposed approach.

In this section, we present our approach for cancer diagnosis using binarilized RNA-seq data. The approach consists of four parts: data binarilization, feature selection, model construction, and model explanation. First, raw RNA-seq data are binarlized. Then, univariate feature selection is applied to select the most relevant features from binarlized data. Processed data are then randomly splitted into a training set and a test set. The training set is used for both hyperparameter search and model training; whereas the test set is used for measuring the performance of trained models. The train-test process is repeated 10 times to get the average results. Models with the highest F1 scores is used to generate SHAP plots. And finally, SHapley Additive exPlanations is used to interpret trained models and determine most impactful features. Fig 1 demonstrates the flow of our proposed approach.

### Data collection

All data used in this study came from the National Cancer Institute Genomic Data Commons (GDC). *Log*_2_(*x* + 1) normalized illumina Hi-Seq RNA sequencing data was merged with clinical information based on their corresponding sample IDs. Samples without RNA-seq information were removed. Samples with primary, recurrent or metastatic tumor were considered as positive samples, while samples with solid tissue standard samples were considered as negative samples. In this study, only RNA-seq data were used, each of which contains the same 20,530 predictors. The properties of each dataset can be found in Table 1.

**Table 1:** Dataset properties.

Name	Total	Positive	Negative
Liver	422	372	50
Lung	1099	989	110
Prostate	550	498	52
Thyroid	572	513	59

All four datasets were normalized using the min-max normalization technique before further processing. All positive samples were labeled as 1, whereas negatives were labeled as 0. After completing all processing, each dataset was divided into a training set and a test set using stratified split. The ratio between a training set and a test set was 80:20.

### Data binarilization

Data binarilization can be seen as an extension of data discretization. Data discretization is the process of converting a feature of continuous values into a finite set of intervals, with each interval representing a range of original values. Although data discretization can reduce the complexity of a dataset, it does not make any feature more interpretable as each feature still contains more than two values. Data binarilization, on the other hand, creates a series of binary features for each continuous feature, with each binary feature representing a range of original values. For each set of binary features, each sample shall have only one positive value, representing the range of values the sample belongs to using the original continuous feature. Because of this characteristic, binarilization offers a more direct view than discretization on the relationships between features and outcomes generated by feature selection and model explanation methods.

A data binarilization tool can be constructed using Algorithm 1.

**Algorithm 1** Data Binarilization Algorithm

**Require**: Dataset *D*, *I* samples, *J* features and *K* binary features

 **function** Data Binarilization(*D*, *K*)

  *BinarilizedDataset* ← ∅

  **for** *j* ← 0, *J* **do**

   **for** *k* ← 0, *K* **do**

    *TempSet* ← ∅

    **for** *i* ← 0, *I* **do**

     **if** $D_{ij}\geq \frac{k}{K}\wedge D_{ij}<\frac{k+1}{K}$ **then**

      *TempSet*_*i*_ ← 1

     **else**

      *TempSet*_*i*_ ← 0

     **end if**

    **end for**

    *BinarilizedDataset* ← *BinarilizedDataset* ∪ *TempSet*

   **end for**

  **end for**

  **return** *BinarilizedDataset*

**end function**

### Feature selection

Because of the high dimensional nature of RNA-seq data and the additional dimensions created by the binarilization process, feature selection was used to remove irrelevant features to reduce overfitting and increase efficiency [34]. In this study, the Chi-Square test (Chi2) was selected. Chi2 is a statistical test primarily used to determine the level of dependency between two categorical variables. For each feature in the feature set, the corresponding *χ*^2^ value is calculated by Eq 1. All features are then ranked in descending order according to their corresponding *χ*^2^ values, with higher *χ*^2^ value indicating higher dependency between the feature and the label. Top-*k* features with the highest *χ*^2^ values are selected to form the reduced feature set, where *k* is the number of features chosen by the user. In this study, we chose 20, 200, 2000, 20000 features, representing 0.01%, 0.1%, 1%, and 10% of binarilized features.

$$\begin{array}{c} \chi^{2}=\sum_{i=1}^{m}\sum_{j=1}^{n}\frac{{\left(O_{ij}-E_{ij}\right)}^{2}}{E_{ij}} \end{array}$$ (1)

In the above formula, *m* denotes the number of features in the feature set, *n* denotes the number of distinct labels, *O*_*ij*_ denotes the observed frequency of having feature as *i* and label as *j*, and *E*_*ij*_ denotes the expected frequency of having feature as *i* and label as *j*.

### Classification model

In order to examine the effectiveness of our proposed data processing technique, we chose to compare the performance of five models based on different algorithms, namely neural network, decision tree, random forest, XGBoost, and support vector machine.

#### Neural network

Neural Network (NN) essentially is just a stack of interconnected layers of nodes. The typical structure of a NN consists of an input layer, one or more than one hidden layers, and an output layer. Each node in the hidden and output layers is connected to all nodes in the previous layer [19]. Each connection has a associated weight. An activation function is also attached to each node in all but the input layer. The activation of an node is determined by the activation function, which calculates the activated value of that node base on all incoming connections. During the training process, the model adjusts all connections’ weights to correctly predict the label of the input data. Sometimes, a dropout rate is also attached to each hidden layer to increase the generalizability of the model. Dropout is a process that reduces the overfitting problem by randomly deactivating some nodes in the hidden layers during the training process. Comparing to other ML algorithms, NN has the advantage of being able to detect the non-linear relationships between input data and output labels. However, NN is computationally more expensive than other algorithms, which limits its applicable areas.

#### Decision tree

Decision tree (DT) is a powerful ML algorithm that is widely used [35]. A DT is made of two types of nodes and one-directional links. Each internal (non-leaf) node represents a test on an attribute, each link represents an outcome of the test, and each terminal (leaf) node represents a class label [36]. Comparing to other algorithms, it is more interpretable because of the tree-like structure, which can be easily converted to decision rules. The tree strcuture is also ideal for capturing interactions between features [37].

#### Random forest

The random forest (RF) algorithm is a decision tree-based bagging ensemble algorithm [38]. Comparing to other algorithms, RF is more robust against noise in data, has higher scalability, and offers strong performance in high-dimensional settings [39]. A random forest classifier generates multiple decision trees. Since not every tree uses all available features or samples, prediction made by each tree is different. Predictions from all trees are then collected and the label that most trees predict will be the final prediction. This process is called the majority voting process.

#### XGBoost

XGBoost is a decision tree-based boosting ensemble learning algorithm. Comparing to all previous boosting tree algorithms, XGBoost offers higher performance and efficiency [40]. Like RF, a XGBoost classifier also generates multiple decision trees. However, unlike bagging ensemble algorithms, boosting ensemble algorithms like XGBoost utilizes a iterative approach to train each sub model, then sequentially combine all sub models to form the final model. The weights of misclassified data in one model will be increased in the next model. Because of this iterative approach, boosting ensemble algorithms are more prone to the overfitting problem.

#### Support vector machine

Support vector machine is a classic ML algorithm for both linear and nonlinear data. In a classification problem, SVM searches for a decision boundary called hyperplane that classify input samples into one of two classes. To achieve this, SVM utilizes kernels to create a feature space in a higher dimension where linear separation is possible. Therefore, SVM is sensitive to kernel choice. SVM is considered highly accurate and less likely to overfit. However, SVM is less efficient than other algorithms [19].

### Hyperparameter search

In order to prevent overfitting and gain maximum performance, hyperparameter search was conducted for each model before training. Because of the lack of knowledge in determining the optimal values for some hyperparameters, random search was used in this study. In this study, each model had 200 variations; the value of each searched hyperparameter of each variation was picked randomly. Based on the F-Measure of each variation, the best performing one was chosen as the predictive model. Due to the small sample sizes of our datasets, 10-fold cross-validation was used in the hyperparameter search processes to avoid over/under-fitting [41]. Tables 2 to 6 contains the searched range for each hyperparameter for each algorithm.

**Table 2:** NN hyperparameters search range.

Name	Value Range
Number of layers	1, 2, 3
Number of nodes per layer	2—input size
Dropout rate	0.1—0.3
Activation Function	ReLU, ELU, GELU, Swish
Learning rate	1
Optimizer	Adadelta

**Table 3:** DT hyperparameters search range.

Name	Value Range
Criterion	gini, entropy
Max number of levels	10—100
Min samples split	2—10
Min samples leaf	1—4
Max features	sqrt, log2

**Table 4:** RF hyperparameters search range.

Name	Value Range
Number of trees	100—1000
Criterion	gini, entropy
Max number of levels	10—100
Min samples split	2—10
Min samples leaf	1—4
Max features	sqrt, log2

**Table 5:** XGBoost hyperparameters search range.

Name	Value Range
Number of trees	100—1000
Max number of levels	10—100
Learning rate	0.01—0.1
Gamma	0.01—0.1
Mininal child weight	0.01—0.1
Lambda	0.01—0.1

**Table 6:** SVM hyperparameters search range.

Name	Value Range
Kernel	Linear, Poly, RBF, Sigmoid

### Evaluation metrics

After constructing and training the prediction models, we used the test sets to evaluate their performance. Because our datasets were imbalanced, metrics designed for imbalanced classification tasks. Specifically, we chose geometric mean (GMean), Matthews correlation coefficient (MCC), F-Measure (F1), and area under the receiver operating characteristic curve (AUC) as our metrics [42–44]. We also included accuracy (ACC) as a metric although it is not particularly suitable for imbalanced classification tasks.

**Accuracy**: measures how many predictions are correct. It is calculated by dividing the number of correct predictions from the total number of predictions made, as shown in Eq 2.

$$\begin{array}{c} Accuracy=\frac{T_{p}+T_{n}}{T_{p}+F_{p}+F_{n}+T_{n}} \end{array}$$ (2)

**Sensitivity**: measures how well the positive class was predicted by calculating the positive rate, as shown in Eq 3.

$$\begin{array}{c} Sensitivity=\frac{T_{p}}{T_{p}+F_{n}} \end{array}$$ (3)

**Specificity**: measures how well the negative class was predicted by calculating the negative rate, as shown in Eq 4.

$$\begin{array}{c} Specificity=\frac{T_{n}}{T_{n}+F_{p}} \end{array}$$ (4)

**Geometric Mean**: is the squared root of the product of the sensitivity and specificity. It is calculated by Eq 5.

$$\begin{array}{c} Geometric\;Mean=\sqrt{Sensitivity*Specificity} \end{array}$$ (5)

**Matthews correlation coefficient**: calculates the *Pearsonproduct* − *momentcorrelationcoefficient* between correct and predicted values [10.1186/s12864-019-6413-7], as shown in Eq 6.

$$\begin{array}{c} MCC=\frac{T_{p}*T_{n}-F_{p}*F_{n}}{\sqrt{\left(T_{p}+F_{p}\right)*\left(T_{p}+F_{n}\right)*\left(T_{n}+F_{p}\right)*\left(T_{n}+F_{n}\right)}} \end{array}$$ (6)

**Precision**: measures the rate of correctly predicted positive samples, as shown in Eq 7.

$$\begin{array}{c} Precision=\frac{T_{p}}{T_{p}+F_{p}} \end{array}$$ (7)

**Recall**: is calculated the same way as sensitivity.

**F-Measure**: is the harmonic mean of precision and recall. It is calculated by Eq 8.

$$\begin{array}{c} F-Measure=\frac{2*Precision*Recall}{Precision+Recall} \end{array}$$ (8)

**Area under the receiver operating characteristic curve**: measures the entire area underneath the receiver operating characteristic curve. It can be calculated by Eq 9.

$$\begin{array}{c} AUC=\frac{S_{p}+n_{p}\left(n_{n}+1\right)/2}{n_{p}*n_{n}} \end{array}$$ (9)

In the above formulas, *T*_*p*_ denotes the number of correctly predicted positive samples, *T*_*n*_ denotes the number of correctly predicted negative samples, *F*_*p*_ denotes the number of incorrectly predicted positive samples, *F*_*n*_ and denotes the number of incorrectly predicted negative samples. For AUC, *S*_*p*_ denotes the sum of the ranks of all positive samples, whereas *n*_*p*_ and *n*_*n*_ denote the number of positive and negative samples respectively.

### Model explanation

Being a black box algorithm, interpreting a random forest is difficult due to the lack of built-in feature importance. To address this issue, we decided to use a popular post-hoc explainer called SHapley Additive exPlanations (SHAP) to explain the relevance of each feature. SHAP is built on basis of game theory concepts, specifically the shapley value. Shapley values are based on the idea that the outcome of a prediction should determine the importance of each feature involved. SHAP utilizes this idea by constructing multiple models with the same set of hyperparameters and training data, but different sets of features. After models are created, the marginal contribution of each feature is calculated by finding the difference between 1) the difference between the prediction of a model with that feature and the average prediction and 2) the difference between the prediction of a model without that feature and the average prediction. Then, SHAP value for each feature is calculated by taking the average of all marginal contributions of that feature [25]. In this study, we chose SHAP because it is able to offer a globally consistent explanation [45]. Specifically, we chose to use TreeExplainer that is built to explain ensemble tree models [46].

## Experiments

### Environmental setup

Program built for this study ran on machines in the Sun Lab at the Penn State Harrisburg. The machines in the Sun Lab ran on Intel(R) Xeon(R) W-2245 CPU @ 3.90GHz. It provided a RAM of size 128 GB. Our study was implemented using Python 3.10.6. Imbalanced-learn 0.9.1 was used for the implementation of GMean metric. Scikit-learn 1.2.2 was used for implementing Chi-Square feature selection, hyperparameter search, three classifiers including decision tree, random forest, and support vector machine, and all other metrics. Shap 0.41.0 was used for the implementation of the SHAP explainer. TensorFlow 2.12.0 was used for building the neural network classifier. And xgboost 1.7.3 was used for implementing the xgboost classifier.

### Performance of models

We compared the performance of models using our proposed data processing technique. To examine how feature selection will affect models that use binarilized data, we ran several experiments with different number of binary features selected. The results of all experiments can be found from Tables 7 to 10. By examining Tables 9 and 10, we can see that the performance of DT is slightly worse than other methods, which is to be expected as other methods theoretically should perform better than DT because of their complexity. Overall, the increase in number of features didn’t affect the performance of models by a large margin.

**Table 7:** Performance for liver data.

Model	Size	Acc	GMean	MCC	F1	AUC
NN	20	0.9824	0.9824	0.9166	0.9824	0.9683
	200	0.9847	0.9847	0.929	0.9847	0.9783
	2000	0.9812	0.9812	0.9094	0.9812	0.9503
DT	20	0.9694	0.9694	0.8487	0.9694	0.9133
	200	0.9624	0.9624	0.8119	0.9624	0.892
	2000	0.9518	0.9518	0.7581	0.9518	0.847
RF	20	0.9882	0.9882	0.9471	0.9882	0.9993
	200	0.9765	0.9765	0.8867	0.9765	0.9433
	2000	0.9647	0.9647	0.8237	0.9647	0.8933
XGBoost	20	0.9765	0.9765	0.8867	0.9765	0.9433
	200	0.9647	0.9647	0.8237	0.9647	0.8933
	2000	0.9647	0.9647	0.8237	0.9647	0.8933
SVM	20	0.9882	0.9882	0.9471	0.9882	0.9993
	200	0.9882	0.9882	0.9471	0.9882	0.9933
	2000	0.9765	0.9765	0.8867	0.9765	0.9433

**Table 8:** Performance for lung data.

Model	Size	Acc	GMean	MCC	F1	AUC
NN	20	0.9912	0.9912	0.9527	0.9912	0.9951
	200	0.9951	0.9951	0.9735	0.9951	0.9973
	2000	0.992	0.992	0.9573	0.992	0.9956
DT	20	0.9947	0.9947	0.9713	0.9947	0.995
	200	0.9858	0.9858	0.9197	0.9858	0.9617
	2000	0.9814	0.9814	0.8936	0.9814	0.9451
RF	20	0.9956	0.9956	0.9756	0.9956	0.9975
	200	0.9912	0.9912	0.9527	0.9912	0.9951
	2000	0.9956	0.9956	0.9756	0.9956	0.9975
XGBoost	20	0.9912	0.9912	0.9527	0.9912	0.9951
	200	0.9912	0.9912	0.9496	0.9912	0.9748
	2000	0.9912	0.9912	0.9527	0.9912	0.9951
SVM	20	0.9867	0.9867	0.9312	0.9867	0.9926
	200	0.9956	0.9956	0.9756	0.9956	0.9975
	2000	1.0	1.0	1.0	1.0	1.0

**Table 9:** Performance for prostate data.

Model	Size	Acc	GMean	MCC	F1	AUC
NN	20	0.9536	0.9536	0.7318	0.9536	0.8755
	200	0.9518	0.9518	0.7389	0.9518	0.9015
	2000	0.9591	0.9591	0.7436	0.9591	0.856
DT	20	0.9509	0.9509	0.7322	0.9509	0.883
	200	0.9364	0.9364	0.6393	0.9364	0.8345
	2000	0.9218	0.9218	0.5625	0.9218	0.786
RF	20	0.9909	0.9909	0.944	0.9909	0.95
	200	0.9545	0.9545	0.7132	0.9545	0.84
	2000	0.9727	0.9727	0.8286	0.9727	0.895
XGBoost	20	0.9636	0.9636	0.8023	0.9636	0.935
	200	0.9545	0.9545	0.7379	0.9545	0.885
	2000	0.9455	0.9455	0.6421	0.9455	0.79
SVM	20	0.9545	0.9545	0.7132	0.9545	0.84
	200	0.9636	0.9636	1.0	0.9636	0.845
	2000	0.9727	0.9727	0.8286	0.9474	0.895

**Table 10:** Performance for thyroid data.

Model	Size	Acc	GMean	MCC	F1	AUC
NN	20	0.9817	0.9817	0.9039	0.9817	0.9567
	200	0.9965	0.9965	0.9815	0.9965	0.987
	2000	0.9965	0.9965	0.9818	0.9965	0.9907
DT	20	0.9696	0.9696	0.8344	0.9696	0.9057
	200	0.9409	0.9409	0.6492	0.9409	0.7903
	2000	0.9417	0.9417	0.6579	0.9417	0.7981
RF	20	0.9652	0.9652	0.8032	0.9652	0.8701
	200	0.9826	0.9826	0.9041	0.9826	0.9167
	2000	0.9652	0.9652	0.8011	0.9652	0.8333
XGBoost	20	0.9739	0.9739	0.8561	0.9739	0.9118
	200	0.9913	0.9913	0.9528	0.9913	0.9583
	2000	1.0	1.0	1.0	1.0	1.0
SVM	20	0.9913	0.9913	0.9561	0.9913	0.9951
	200	0.9739	0.9739	0.8561	0.9739	0.9118
	2000	0.9913	0.9913	0.9561	0.9913	0.9951

We also compare the performance of models using our proposed data binarilization technique with other models based on other feature selection or dimensionality reduction techniques. By examining Tables 11 to 14, we can see that models using our proposed technique, despite having to rely on less features because of binarilization, perform about the same as models using other techniques. This proves that not only certain genes, but also some value ranges of certain genes are not relevant to cancer prediction.

**Table 11:** Performance for 20-feature liver data.

Model	Method	Acc	GMean	MCC	F1	AUC
NN	AE	0.9729	0.9729	0.865	0.9729	0.924
	Chi2	0.98	0.98	0.9048	0.98	0.9583
	PCA	0.9871	0.9871	0.941	0.9871	0.9883
	Hybrid	0.9882	0.9882	0.9471	0.9882	0.9933
	Proposed	0.9824	0.9824	0.9166	0.9824	0.9683
DT	AE	0.9624	0.9624	0.8089	0.9624	0.8833
	Chi2	0.9588	0.9588	0.7879	0.9588	0.8553
	PCA	0.9518	0.9518	0.7579	0.9518	0.847
	Hybrid	0.9694	0.9694	0.8511	0.9694	0.9263
	Proposed	0.9694	0.9694	0.8487	0.9694	0.9133
RF	AE	0.9647	0.9647	0.8237	0.9647	0.8933
	Chi2	0.9647	0.9647	0.8237	0.9647	0.8933
	PCA	0.9882	0.9882	0.9424	0.9882	0.95
	Hybrid	0.9882	0.9882	0.9471	0.9882	0.9933
	Proposed	0.9882	0.9882	0.9471	0.9882	0.9993
XGBoost	AE	0.9529	0.9529	0.7577	0.9529	0.8433
	Chi2	0.9647	0.9647	0.8237	0.9647	0.8933
	PCA	0.9882	0.9882	0.9471	0.9882	0.9933
	Hybrid	0.9882	0.9882	0.9471	0.9882	0.9933
	Proposed	0.9765	0.9765	0.8867	0.9765	0.9433
SVM	AE	0.9882	0.9882	0.9471	0.9882	0.9933
	Chi2	0.9765	0.9765	0.8867	0.9765	0.9433
	PCA	0.9882	0.9882	0.9471	0.9882	0.9933
	Hybrid	0.9882	0.9882	0.9471	0.9882	0.9933
	Proposed	0.9882	0.9882	0.9471	0.9882	0.9993

**Table 12:** Performance for 20-feature lung data.

Model	Method	Acc	GMean	MCC	F1	AUC
NN	AE	0.9991	0.9991	0.9953	0.9991	0.9995
	Chi2	0.9894	0.9894	0.9403	0.9894	0.9738
	PCA	0.9889	0.9889	0.8928	0.9889	0.9493
	Hybrid	0.9982	0.9982	0.9901	0.9982	0.997
	Proposed	0.9536	0.9536	0.7318	0.9536	0.8755
DT	AE	0.9956	0.9956	0.9802	0.9956	0.994
	Chi2	0.9845	0.9845	0.9151	0.9845	0.9691
	PCA	0.9881	0.9881	0.9361	0.9881	0.9832
	Hybrid	0.9898	0.9898	0.946	0.9898	0.9842
	Proposed	0.9947	0.9947	0.9713	0.9947	0.995
RF	AE	0.9956	0.9956	0.9756	0.9956	0.9975
	Chi2	0.9867	0.9867	0.9262	0.9867	0.9724
	PCA	0.9956	0.9956	0.9756	0.9956	0.9975
	Hybrid	1.0	1.0	1.0	1.0	1.0
	Proposed	0.9956	0.9956	0.9756	0.9956	0.9975
XGBoost	AE	1.0	1.0	1.0	1.0	1.0
	Chi2	0.9965	0.9965	0.9746	0.9965	0.9773
	PCA	0.9956	0.9956	0.9756	0.9956	0.9975
	Hybrid	0.9912	0.9912	0.9496	0.9912	0.9748
	Proposed	0.9912	0.9912	0.9527	0.9912	0.9951
SVM	AE	1.0	1.0	1.0	1.0	1.0
	Chi2	0.9867	0.9867	0.9262	0.9867	0.9724
	PCA	0.9956	0.9956	0.9756	0.9956	0.9975
	Hybrid	0.9912	0.9912	0.9527	0.9912	0.9951
	Proposed	0.9867	0.9867	0.9312	0.9867	0.9926

**Table 13:** Performance for 20-feature prostate data.

Model	Method	Acc	GMean	MCC	F1	AUC
NN	AE	0.9718	0.9718	0.8261	0.9718	0.899
	Chi2	0.9436	0.9436	0.6647	0.9436	0.8385
	PCA	0.96	0.96	0.7604	0.96	0.8835
	Hybrid	0.9491	0.9491	0.7237	0.9491	0.8955
	Proposed	0.9491	0.9491	0.7187	0.9491	0.882
DT	AE	0.9409	0.9409	0.6395	0.9409	0.8145
	Chi2	0.9082	0.9082	0.4919	0.9082	0.765
	PCA	0.9218	0.9218	0.5627	0.9218	0.795
	Hybrid	0.9145	0.9145	0.525	0.9145	0.7775
	Proposed	0.9509	0.9509	0.7322	0.9509	0.883
RF	AE	0.9636	0.9636	0.7638	0.9636	0.845
	Chi2	0.9545	0.9545	0.7132	0.9545	0.84
	PCA	0.9636	0.9636	0.7638	0.9636	0.845
	Hybrid	0.9545	0.9545	0.7379	0.9545	0.885
	Proposed	0.9909	0.9909	0.944	0.9909	0.95
XGBoost	AE	0.9636	0.9636	0.78	0.9636	0.89
	Chi2	0.9	0.9	0.5194	0.9	0.81
	PCA	0.9273	0.9273	0.4811	0.9273	0.69
	Hybrid	0.9364	0.9364	0.5653	0.9364	0.74
	Proposed	0.9636	0.9636	0.8023	0.9636	0.935
SVM	AE	0.9636	0.9636	0.78	0.9636	0.89
	Chi2	0.9091	0.9091	0.45	0.9091	0.725
	PCA	0.9636	0.9636	0.78	0.9636	0.89
	Hybrid	0.9545	0.9545	0.7132	0.9545	0.84
	Proposed	0.9545	0.9545	0.7132	0.9545	0.84

**Table 14:** Performance for 20-feature thyroid data.

Model	Method	Acc	GMean	MCC	F1	AUC
NN	AE	0.987	0.987	0.9335	0.987	0.978
	Chi2	0.9765	0.9765	0.8719	0.9765	0.9206
	PCA	0.9713	0.9713	0.8561	0.9713	0.9472
	Hybrid	0.9809	0.9809	0.8988	0.9809	0.9525
	Proposed	0.9817	0.9817	0.9039	0.9817	0.9567
DT	AE	0.9435	0.9435	0.6784	0.9435	0.8102
	Chi2	0.9565	0.9565	0.7727	0.9565	0.8911
	PCA	0.9348	0.9348	0.6354	0.9348	0.7943
	Hybrid	0.9496	0.9496	0.7092	0.9496	0.8356
	Proposed	0.9696	0.9696	0.8344	0.9696	0.9057
RF	AE	0.9478	0.9478	0.6893	0.9478	0.7868
	Chi2	0.9826	0.9826	0.907	0.9826	0.9535
	PCA	0.9565	0.9565	0.7478	0.9565	0.8285
	Hybrid	0.9826	0.9826	0.9041	0.9826	0.9167
	Proposed	0.9652	0.9652	0.8032	0.9652	0.8701
XGBoost	AE	0.9565	0.9565	0.7478	0.9565	0.8285
	Chi2	0.9565	0.9565	0.7594	0.9565	0.8653
	PCA	0.9826	0.9826	0.9041	0.9826	0.9167
	Hybrid	0.9739	0.9739	0.8663	0.9739	0.9486
	Proposed	0.9739	0.9739	0.8561	0.9739	0.9118
SVM	AE	1.0	1.0	1.0	1.0	1.0
	Chi2	0.9739	0.9739	0.8561	0.9739	0.9118
	PCA	0.9913	0.9913	0.9528	0.9913	0.9583
	Hybrid	0.9826	0.9826	0.907	0.9826	0.9535
	Proposed	0.9913	0.9913	0.9561	0.9913	0.9951

### Explanation results

**Fig 2:**
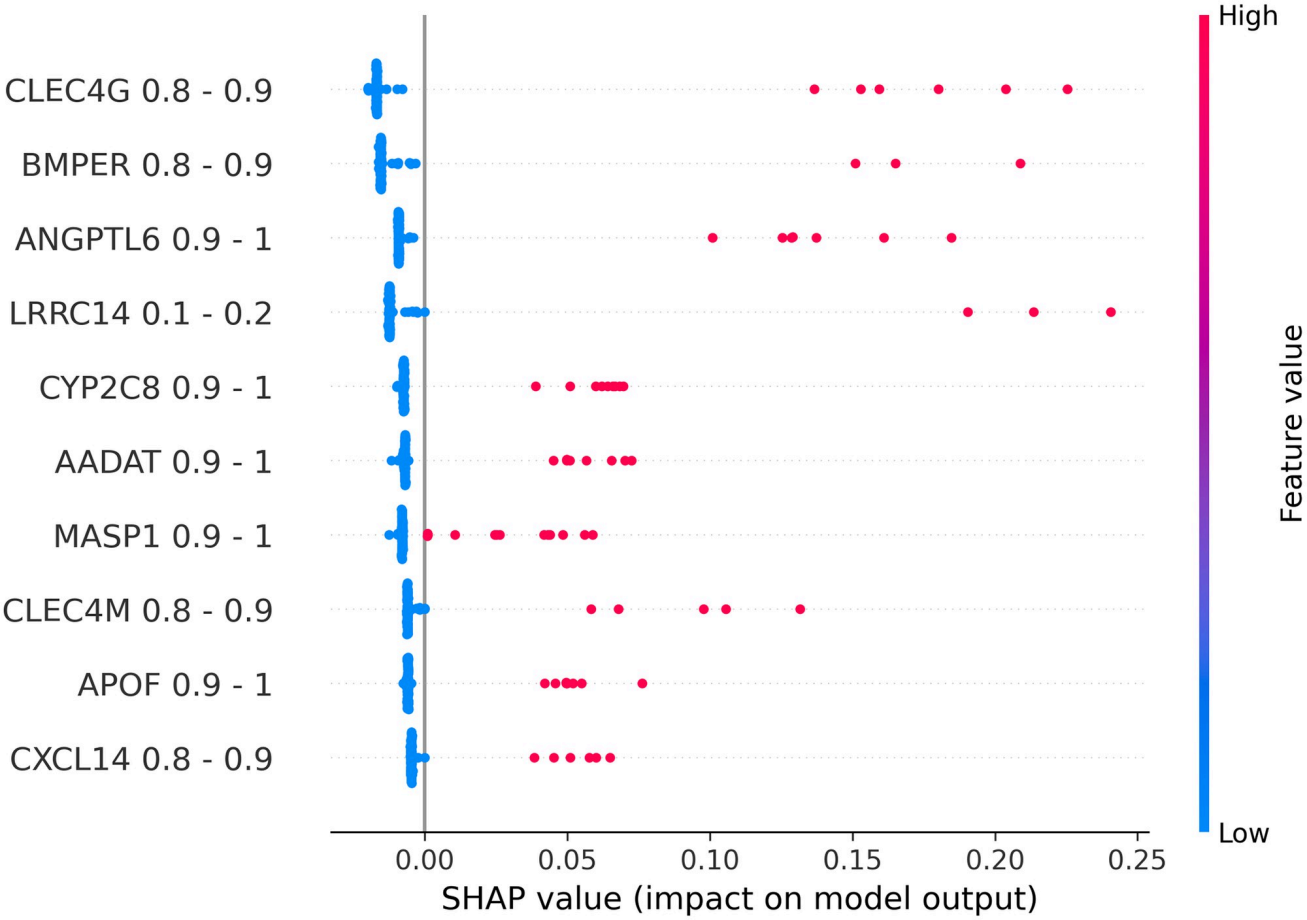
Shap plots for 20-feature liver data.

**Fig 3:**
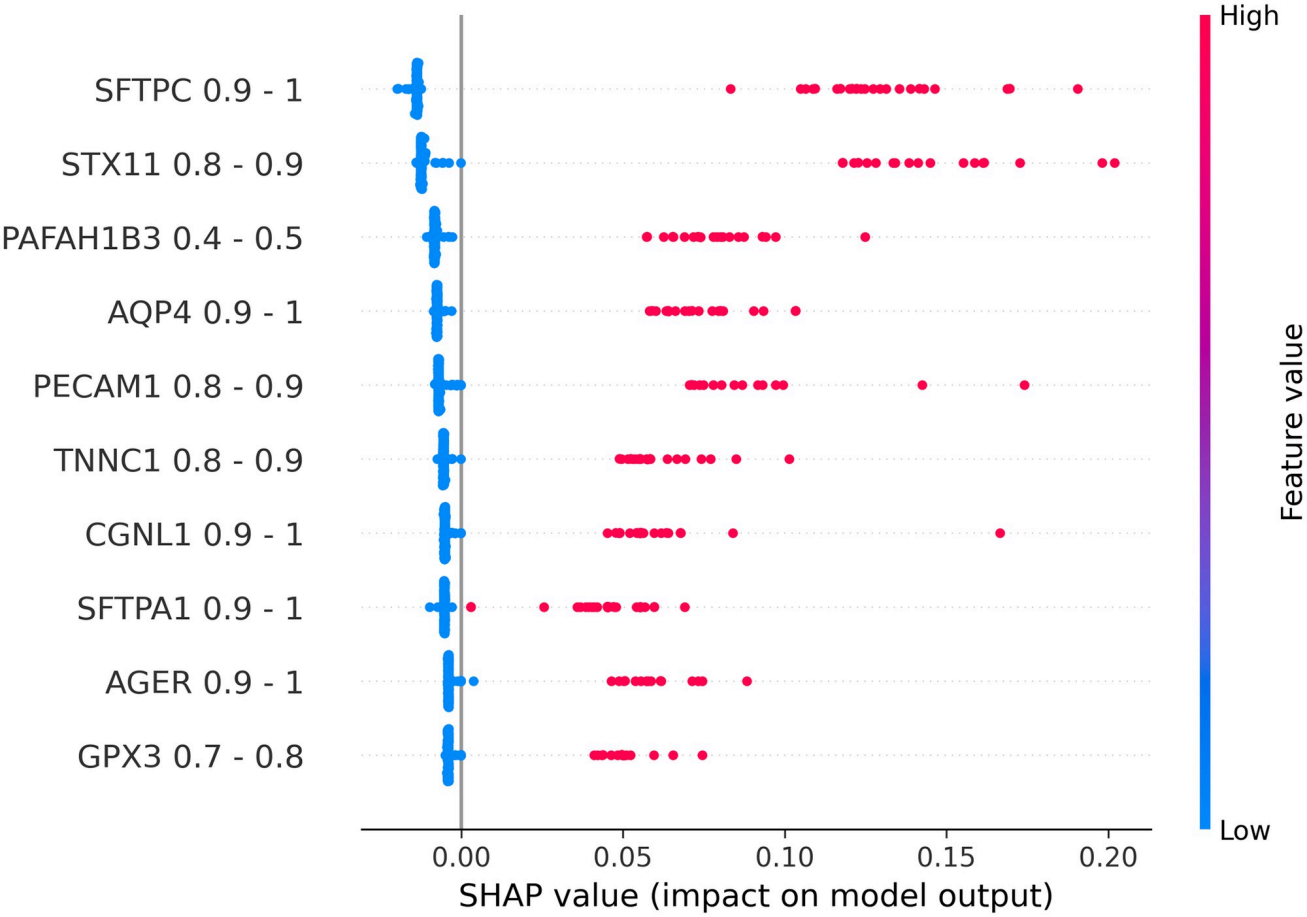
Shap plots for 20-feature lung data.

**Fig 4:**
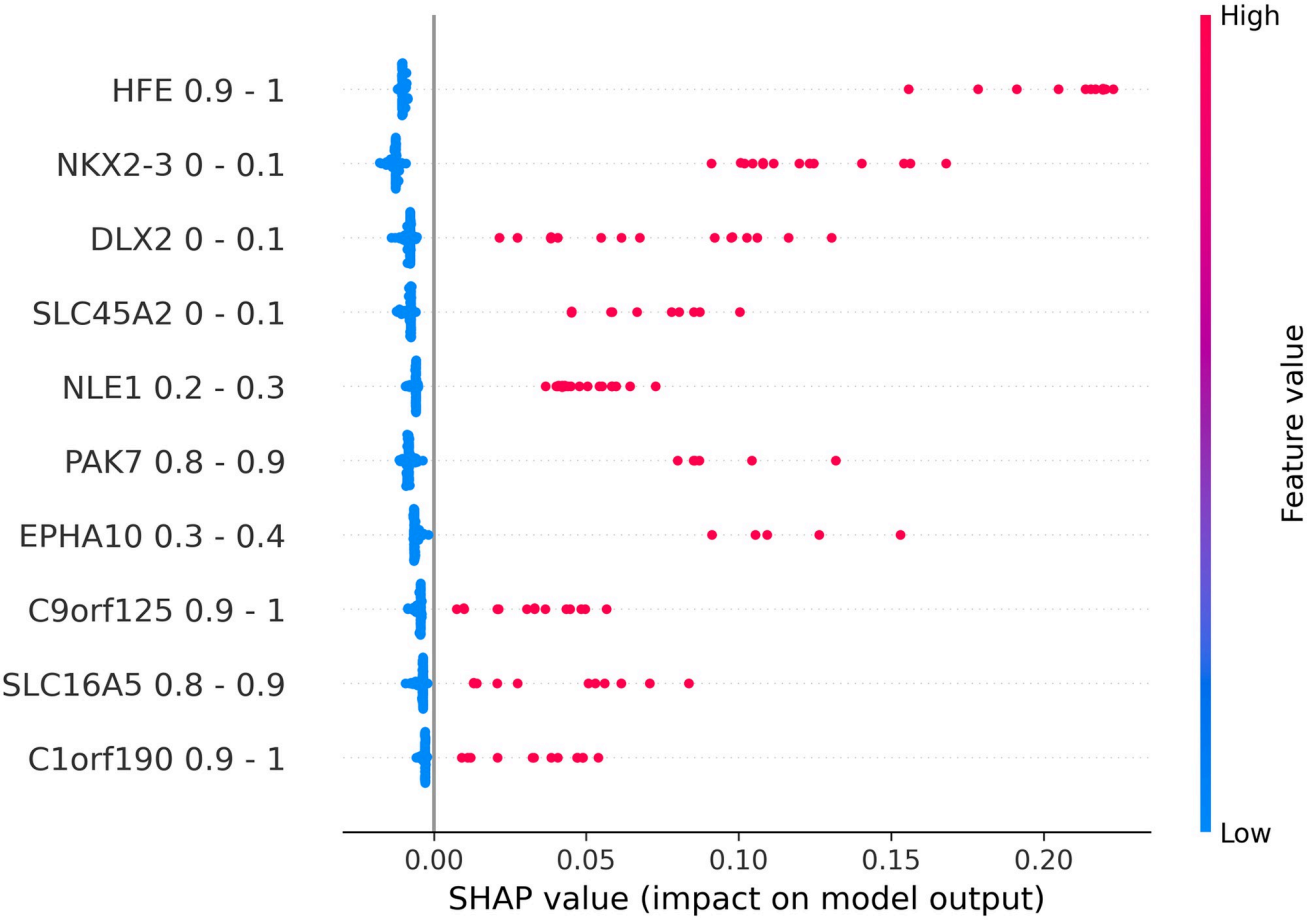
Shap plots for 20-feature prostate data.

**Fig 5:**
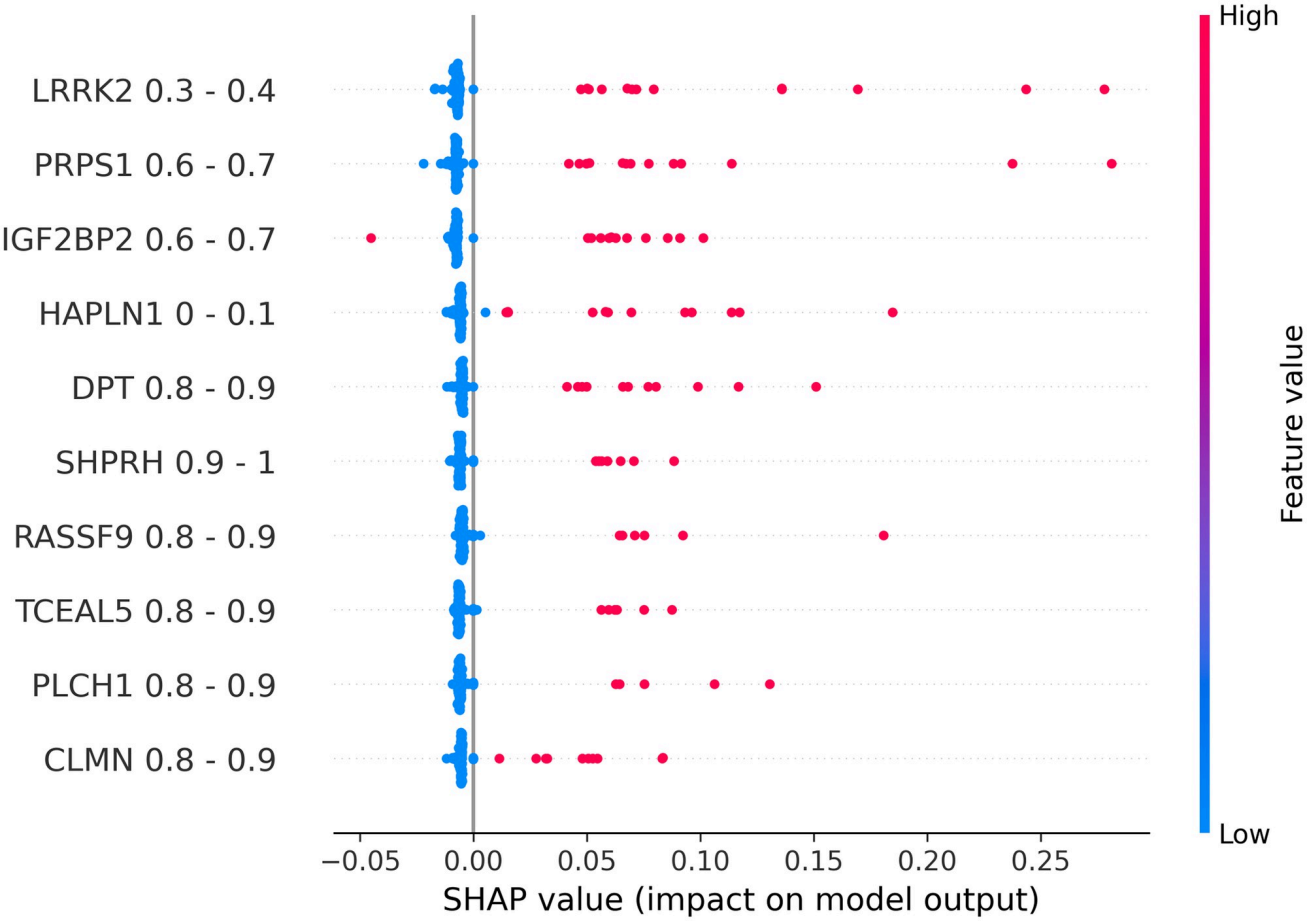
Shap plots for 20-feature thyroid data.

To explain the relationship between input features and output labels, SHAP beeswarm plots were generated to visualize such relationships for each model. In a beeswarm plot, the feature names on the left side are ranked from top to bottom base on their corresponding absolute SHAP values. The horizontal axis represents the SHAP value of each data point. In places where multiple data points share the same SHAP value, dots are stacked vertically. The line at the 0 point separates samples that negatively contribute to the prediction from ones that have a positive contribution. We can see that, because our test data was imbalanced, the number of samples on the left side of the 0-line is significantly smaller than the one on the right side. The colorbar on the right side represents the value of the corresponding feature of each data point. Based on these properties, we can see that data binarilization gives a more direct view on the relationships between each gene and the predicted outcome by examining Figs 2–5, being able to show not only the most impactful features, but also their associated value ranges. Since each binary feature can only be either 0 or 1, SHAP plots for binarlized data only has two colors representing feature values.

## Discussion

In this study, we propose a novel data processing technique for cancer-related RNA-seq data. After binarilization, each gene is splitted into ten binary features. For each sample only one of the ten binary features is positive, indicating the range of values the sample’s original value of that feature lies between. Because of data binarilization will increase the number of features, we performed feature selection to filter out irrelevant features. We compared the performance of models using binarilized features with models using continuous features. The results show that data binarilization does not affect the predictive performance of models. For model explanation, we used SHAP to rank all features in terms of relevance to prediction. Comparing to other explanation models that use continuous data like iAFPs-EnC-GA, AIPs-SnTCN, and OncoNetExplainer, using binarlized data makes understanding results of SHAP analysis easier because the relevant features along with the relevant value range of the feature are revealed together.

Although we presented a novel approach that shows promising results, there are still some limitations to this study. First, the number of samples in each dataset was quite small. Thus, this work represents a proof-of-concept study for the data binarilization approach. In future, we plan to apply this approach to larger datasets to examine its effectiveness. Second, the datasets were highly imbalanced. Although there are several imbalance treatment options like oversampling and undersampling, we decided not to use them as oversampling could create invalid samples that has more than one binary feature that belong to the same original feature be positive, whereas undersampling will further decrease the number of samples. Therefore, we included metrics designed for imbalance classification tasks to mitigate this issue. Third, because some of the models included in this study were black box models, SHAP was used to provide explanations for them. However, post-hoc explanation techniques like SHAP is prone to biases exist in training data, which could lead to misleading and unfaithful explanations of models. Therefore, researchers suggest that white box models should be developed for fields involve high-stakes decision making [47]. Following such suggestions, we plan to utilize our proposed data processing technique to build more interpretable white box models in our future studies. Last but not least, the hidden properties of the datasets, such as the demography of patients and collection time, could have significant impact on the performance of data-driven techniques like ML models. We plan to collaborate with clinicians to test our proposed approach in the real world in our future studies.

## Conclusion

Early detection of cancer can increase patients’ survival chances [48]. Recent developments in technology have enabled the use of new diagnostic methods like ML-assisted models. Because ML models excel at processing complex data, this allows researchers to utilize high-dimensional data like RNA-seq data to predict cancer patients and extract relevant biomarkers. However, ML models suffer from problems like poor interpretability. In this study, we proposed a novel approach that utilize data binarilization to increase interpretability of ML models for cancer diagnosis. We proved that models using binarlized data can achieve the same level of performance while relying on less features. We also showed that data binarilization offers higher interpretability by offering a direct view on how each feature impacts the outcome. In future, we plan to address some limitations mentioned above by proposing new algorithms and establishing collaborations with clinicians. We also plan to apply this approach to address other healthcare problems.

## References

[1] Cruz JA, Wishart DS. Applications of machine learning in cancer prediction and prognosis. Cancer informatics. 2006 Jan;2:117693510600200030. doi: 10.1177/117693510600200030 PMC2675494 19458758

[2] Sharma A, Rani R. A systematic review of applications of machine learning in cancer prediction and diagnosis. Archives of Computational Methods in Engineering. 2021 Dec;28(7):4875–96. doi: 10.1007/s11831-021-09556-z

[3] Glaab E, Bacardit J, Garibaldi JM, Krasnogor N. Using rule-based machine learning for candidate disease gene prioritization and sample classification of cancer gene expression data. PloS one. 2012 Jul 11;7(7):e39932. doi: 10.1371/journal.pone.0039932 22808075 PMC3394775

[4] Li J, Zhou Z, Dong J, Fu Y, Li Y, Luan Z, et al. Predicting breast cancer 5-year survival using machine learning: A systematic review. PloS one. 2021 Apr 16;16(4):e0250370. doi: 10.1371/journal.pone.0250370 33861809 PMC8051758

[5] Ghaly G, Tallima H, Dabbish E, Badr ElDin N, Abd El-Rahman MK, Ibrahim MA, et al. Anti-Cancer Peptides: Status and Future Prospects. Molecules. 2023 Jan 23;28(3):1148. doi: 10.3390/molecules28031148 36770815 PMC9920184

[6] Hajisharifi Z, Piryaiee M, Beigi MM, Behbahani M, Mohabatkar H. Predicting anticancer peptides with Chou’s pseudo amino acid composition and investigating their mutagenicity via Ames test. Journal of theoretical biology. 2014 Jan 21;341:34–40. doi: 10.1016/j.jtbi.2013.08.037 24035842

[7] Chen W, Ding H, Feng P, Lin H, Chou KC. iACP: a sequence-based tool for identifying anticancer peptides. Oncotarget. 2016 Mar 3;7(13):16895. doi: 10.18632/oncotarget.7815 26942877 PMC4941358

[8] Akbar S, Hayat M, Iqbal M, Jan MA. iACP-GAEnsC: Evolutionary genetic algorithm based ensemble classification of anticancer peptides by utilizing hybrid feature space. Artificial intelligence in medicine. 2017 Jun 1;79:62–70. doi: 10.1016/j.artmed.2017.06.008 28655440

[9] Akbar S, Rahman AU, Hayat M, Sohail M. cACP: Classifying anticancer peptides using discriminative intelligent model via Chou’s 5-step rules and general pseudo components. Chemometrics and Intelligent Laboratory Systems. 2020 Jan 15;196:103912. doi: 10.1016/j.chemolab.2019.103912

[10] Akbar S, Hayat M, Tahir M, Chong KT. cACP-2LFS: classification of anticancer peptides using sequential discriminative model of KSAAP and two-level feature selection approach. IEEE Access. 2020 Jul 14;8:131939–48. doi: 10.1109/ACCESS.2020.3009125

[11] Akbar S, Hayat M, Tahir M, Khan S, Alarfaj FK. cACP-DeepGram: classification of anticancer peptides via deep neural network and skip-gram-based word embedding model. Artificial intelligence in medicine. 2022 Sep 1;131:102349. doi: 10.1016/j.artmed.2022.102349 36100346

[12] Danaee P, Ghaeini R, Hendrix DA. A deep learning approach for cancer detection and relevant gene identification. In Pacific symposium on biocomputing 2017 2017 (pp. 219-229). 10.1142/9789813207813_0022 PMC5177447 27896977

[13] Vanitha CD, Devaraj D, Venkatesulu I. Gene expression data classification using support vector machine and mutual information-based gene selection. procedia computer science. 2015 Jan 1;47:13–21. doi: 10.1016/j.procs.2015.03.178

[14] Juneja K, Rana C. An improved weighted decision tree approach for breast cancer prediction. International Journal of Information Technology. 2020 Sep;12(3):797–804. doi: 10.1007/s41870-018-0184-2

[15] Osareh A, Shadgar B. Machine learning techniques to diagnose breast cancer. In 2010 5th international symposium on health informatics and bioinformatics 2010 Apr 20 (pp. 114-120). IEEE.

[16] Liu M, Fan X, Fang K, Zhang Q, Ma S. Integrative sparse principal component analysis of gene expression data. Genetic epidemiology. 2017 Dec;41(8):844–65. doi: 10.1002/gepi.22089 29114920 PMC5912177

[17] Fakoor R, Ladhak F, Nazi A, Huber M. Using deep learning to enhance cancer diagnosis and classification. In Proceedings of the international conference on machine learning 2013 Jun (Vol. 28, pp. 3937-3949). New York, NY, USA: ACM.

[18] Xiao Y, Wu J, Lin Z, Zhao X. A semi-supervised deep learning method based on stacked sparse auto-encoder for cancer prediction using RNA-seq data. Computer methods and programs in biomedicine. 2018 Nov 1;166:99–105. doi: 10.1016/j.cmpb.2018.10.004 30415723

[19] Kabir MF, Chen T, Ludwig SA. A performance analysis of dimensionality reduction algorithms in machine learning models for cancer prediction. Healthcare Analytics. 2023;3:100125. doi: 10.1016/j.health.2022.100125

[20] Dass S, Mistry S, Sarkar P, Barik S, Dahal K. A proficient two stage model for identification of promising gene subset and accurate cancer classification. International Journal of Information Technology. 2023 Mar;15(3):1555–68. doi: 10.1007/s41870-023-01181-2

[21] Zebari R, Abdulazeez A, Zeebaree D, Zebari D, Saeed J. A comprehensive review of dimensionality reduction techniques for feature selection and feature extraction. Journal of Applied Science and Technology Trends. 2020 May 15;1(2):56–70. doi: 10.38094/jastt1224

[22] Björklund A, Mäkelä J, Puolamäki K. SLISEMAP: Supervised dimensionality reduction through local explanations. Machine Learning. 2023 Jan;112(1):1–43. doi: 10.1007/s10994-022-06261-1

[23] Loyola-Gonzalez O. Black-box vs. white-box: Understanding their advantages and weaknesses from a practical point of view. IEEE access. 2019 Oct 24;7:154096–113. doi: 10.1109/ACCESS.2019.2949286

[24] Ribeiro MT, Singh S, Guestrin C. “Why should i trust you?” Explaining the predictions of any classifier. In Proceedings of the 22nd ACM SIGKDD international conference on knowledge discovery and data mining 2016 Aug 13 (pp. 1135-1144).

[25] Lundberg SM, Lee SI. A unified approach to interpreting model predictions. Advances in neural information processing systems. 2017;30.

[26] Schaduangrat N, Nantasenamat C, Prachayasittikul V, Shoombuatong W. ACPred: a computational tool for the prediction and analysis of anticancer peptides. Molecules. 2019 May 22;24(10):1973. doi: 10.3390/molecules24101973 31121946 PMC6571645

[27] Ahmad A, Akbar S, Tahir M, Hayat M, Ali F. iAFPs-EnC-GA: identifying antifungal peptides using sequential and evolutionary descriptors based multi-information fusion and ensemble learning approach. Chemometrics and Intelligent Laboratory Systems. 2022 Mar 15;222:104516. doi: 10.1016/j.chemolab.2022.104516

[28] Raza A, Uddin J, Almuhaimeed A, Akbar S, Zou Q, Ahmad A. AIPs-SnTCN: Predicting anti-inflammatory peptides using fastText and transformer encoder-based hybrid word embedding with self-normalized temporal convolutional networks. Journal of Chemical Information and Modeling. 2023 Oct 31;63(21):6537–54. doi: 10.1021/acs.jcim.3c01563 37905969

[29] Han B, Zhao N, Zeng C, Mu Z, Gong X. ACPred-BMF: bidirectional LSTM with multiple feature representations for explainable anticancer peptide prediction. Scientific Reports. 2022 Dec 19;12(1):21915. doi: 10.1038/s41598-022-24404-1 36535969 PMC9763336

[30] Ramírez-Mena A, Andrés-León E, Alvarez-Cubero MJ, Anguita-Ruiz A, Martinez-Gonzalez LJ, Alcala-Fdez J. Explainable artificial intelligence to predict and identify prostate cancer tissue by gene expression. Computer Methods and Programs in Biomedicine. 2023 Oct 1;240:107719. doi: 10.1016/j.cmpb.2023.107719 37453366

[31] Karim MR, Cochez M, Beyan O, Decker S, Lange C. OncoNetExplainer: explainable predictions of cancer types based on gene expression data. In2019 IEEE 19th International Conference on Bioinformatics and Bioengineering (BIBE) 2019 Oct 28 (pp. 415-422). IEEE.

[32] National Institute of Statistical Sciences (NISS). In: YouTube [Internet]. 17 Oct.2022 [cited 3 Oct.2023]. Available: https://www.youtube.com/watch?v=RaCHTDRKvPk&t=1777s

[33] Wei L, Zhou C, Chen H, Song J, Su R. ACPred-FL: a sequence-based predictor using effective feature representation to improve the prediction of anti-cancer peptides. Bioinformatics. 2018 Dec 1;34(23):4007–16. doi: 10.1093/bioinformatics/bty451 29868903 PMC6247924

[34] Jović A, Brkić K, Bogunović N. A review of feature selection methods with applications. In 2015 38th international convention on information and communication technology, electronics and microelectronics (MIPRO) 2015 May 25 (pp. 1200-1205). Ieee.

[35] Charbuty B, Abdulazeez A. Classification based on decision tree algorithm for machine learning. Journal of Applied Science and Technology Trends. 2021 Mar 24;2(01):20–8. doi: 10.38094/jastt20165

[36] Kabir MF, Ludwig SA. Classification models and survival analysis for prostate cancer using RNA sequencing and clinical data. In2019 IEEE international conference on big data (big data) 2019 Dec 9 (pp. 2736-2745). IEEE.

[37] Molnar C. Interpretable machine learning. Lulu. com; 2020.

[38] Breiman L. Random forests. Machine learning. 2001 Oct;45:5–32. doi: 10.1023/A:1017934522171

[39] Biau G, Scornet E. A random forest guided tour. Test. 2016 Jun;25:197–227. doi: 10.1007/s11749-016-0481-7

[40] Chen T, Guestrin C. Xgboost: A scalable tree boosting system. InProceedings of the 22nd acm sigkdd international conference on knowledge discovery and data mining 2016 Aug 13 (pp. 785-794).

[41] Menden MP, Iorio F, Garnett M, McDermott U, Benes CH, Ballester PJ, et al. Machine learning prediction of cancer cell sensitivity to drugs based on genomic and chemical properties. PLoS one. 2013 Apr 30;8(4):e61318. doi: 10.1371/journal.pone.0061318 23646105 PMC3640019

[42] Guo H, Liu H, Wu C, Zhi W, Xiao Y, She W. Logistic discrimination based on G-mean and F-measure for imbalanced problem. Journal of Intelligent & Fuzzy Systems. 2016 Jan 1;31(3):1155–66. doi: 10.3233/IFS-162150

[43] Chicco D, Jurman G. The advantages of the Matthews correlation coefficient (MCC) over F1 score and accuracy in binary classification evaluation. BMC genomics. 2020 Dec;21(1):1–3. doi: 10.1186/s12864-019-6413-7 31898477 PMC6941312

[44] Gaudreault JG, Branco P, Gama J. An analysis of performance metrics for imbalanced classification. In International Conference on Discovery Science 2021 Oct 9 (pp. 67-77). Cham: Springer International Publishing.

[45] Rathi S. Generating counterfactual and contrastive explanations using SHAP. arXiv preprint arXiv:1906.09293. 2019 Jun 21.

[46] Lundberg SM, Erion G, Chen H, DeGrave A, Prutkin JM, Nair B, et al. From local explanations to global understanding with explainable AI for trees. 10.1038/s42256-019-0138-9 PMC7326367 32607472

[47] Rudin C. Stop explaining black box machine learning models for high stakes decisions and use interpretable models instead. Nature machine intelligence. 2019 May;1(5):206–15. Nature machine intelligence. 2020 Jan;2(1):56-67. doi: 10.1038/s42256-019-0048-x 35603010 PMC9122117

[48] Shi M, Zhang B. Semi-supervised learning improves gene expression-based prediction of cancer recurrence. Bioinformatics. 2011 Nov 1;27(21):3017–23. doi: 10.1093/bioinformatics/btr502 21893520 PMC3198572

