# Supplementary material for: Explainable machine learning approach for cancer prediction through binarilization of RNA sequencing data
Source: PLoS One. 2024 May 10;19(5):e0302947. doi: 10.1371/journal.pone.0302947 (PMC11086842; doi:10.1371/journal.pone.0302947)
Supplement: Attachment — Submitted filename: Response to Reviewer - March 26 2024.docx [file pone.0302947.s001.docx]

**A point-by-point response to the reviewer’s comments and suggestions:**

**Reviewer #1**:

1. At the end of introduction, the authors should add novelty and contributions in points.

**Response**: Thanks to the reviewer for bringing this up. We added a list of contributions at the end of the introduction section accordingly.

2. in the related work section, the authors should discuss the peptide based approaches by citing the recent predictors such as, iACP-GAEnsC, cACP, cACP-2LFS, and cACP-DeepGram for the reader concerns.

**Response**: Thanks to the reviewer for the comment and suggestions. We added a new paragraph to cover the peptide-based approaches.

3. the quality of figures are poor, authors should revised figures in 300dpi.

**Response**: Thanks to the review for bringing this up. We changed all figures in the manuscript to 300 dpi.

4. how the authors handle the overfitting issues of the proposed model.

**Response**: Thanks to the reviewer for the comment. In this study, several techniques are used to prevent overfitting. Cross validation was used during the hyperparameter search process. Because data binarilization will inevitably increase the number of features, Chi-Sqaure feature selection technique was used to reduce the complexity of models used in this study by reducing the number of features. On the model side, dropout layers were added to all neural network models. The random forest algorithm also avoids the overfitting problem through ensemble learning.

5. in SHAP interpretation, the authors are advised to incorporate and discuss the recent predictors such as iAFPs-EnC-GA, and AIPs-SnTCN.

**Response**: Thanks to the reviewer for bringing this up. We added discussions on the recent explainable predictors in our literature review part of the introduction section.

6. what should be the future directions of the proposed model.

**Response**: We appreciate the reviewer for the comment. The future plans for this proposed technique are 1) building inherently interpretable, also known as white box, models based on our proposed technique, 2) examining the effectiveness of our proposed technique on making training machine learning models more efficient, 3) developing a new imbalance treatment technique for our proposed technique, and 4) work with professionals from other fields to create interpretable models to address real world issues.

**Reviewer #2**:

1. To validate the effectiveness of the proposed model, the authors needs a thorough comparison with existing state of the art methods.

**Response**: We appreciate the reviewer for bringing this this up. In addition to the original random forest algorithm, we added four more algorithms, namely neural networks, decision tree, xgboost, and support vector machine. We also added comparison between models using our binarilized data with models using data processed by other state-of-the-art feature selection or dimensionality reduction techniques.

2. What should be real life applications and importance of the proposed model.

**Response**: Thanks to the review for the comment. The main focus of this paper is the examination of the effectiveness of using binarlized RNA-seq data in ML-based cancer prediction models. Since binarilized data only have two values, 0 or 1, it reduces the complexity of the data, which facilitates both processing and interpretation. Since each feature now represents a range of value of an original feature, interpretation of the model becomes easier as the relevant features and its relevant value range can be revealed by using interpretation or explanation techniques. We believe that data binarlization could help build more interpretable models for facilitating high-stakes decision making when many continous variables are involved. We also believe that data binarilization could make training machine learning models more efficient. We will investigate this in our future studies.

3. For clear visualization, the authors should be represented in 300 dpi.

**Response**: Thanks to the review for bringing this up. We changed all figures in the manuscript to 300 dpi.

4. I suggest to incorporate a discussion section to shows the contribution, advantages, limitations, and improvement than existing studies.

**Response**: Thanks to the reviewer for the comment. We changed the original limitation section to a discussion section and added contributions, advantages, and improvements over the existing methods. Because of this addition, the limitations and future plans are now covered in the second paragraph of the discussion section.

5. The recent computational models needs to be cited related to biomedical engineering and ML such as; doi.org/10.3389/fgene.2020.539227, doi.org/10.1016/j.bbe.2020.05.010, and 10.1109/TNSRE.2016.2636367

**Response**: We appreciate the reviewer for the comments and suggestions. We examined the articles the reviewer suggested. However, we believe that these articles are not relevant to our study as our study is focused more on the studying of cancer and RNA sequencing data. Because of this reason, we chose to not include these articles.
